# Cognitive and Composite Behavioural Welfare Assessments of Pet Cats between the Ages of 9–22 Months, Living in Single and Multi-Cat Households

**DOI:** 10.3390/ani11061793

**Published:** 2021-06-16

**Authors:** Sezan Ozgunay, Jane K. Murray, Elizabeth Rowe, Nancy R. Gee, Marije Bartholomeus, Rachel Casey

**Affiliations:** 1 School of Veterinary Science, University of Bristol, Langford, Bristol BS40 5DU, UK; jane.murray@dogstrust.org.uk (J.K.M.); lizzie.rowe88@gmail.com (E.R.); rachel.casey@dogstrust.org.uk (R.C.); 2 Center for Human-Animal Interaction, School of Medicine, Virginia Commonwealth University, Richmond, VA 23298, USA; nancy.gee@vcuhealth.org; 3 Sarphatistraat 81-2, 1018EZ Amsterdam, The Netherlands; marije1985@gmail.com

**Keywords:** judgment bias, cat stress score, measuring welfare, sociality, affective state, domestic cat

## Abstract

**Simple Summary:**

Although agonistic interactions between cats are often regarded clinically as a source of stress, there is currently limited research evidence regarding the welfare impact of keeping multiple cats as pets. The aim of this study was to compare welfare indicators between cats living in single and multi-cat households, as well as between cats living in multi-cat households where agonistic behaviour was/was not reported by owners. Indicators included a spatial judgment bias task (JBT) and the cat stress score (CSS). CSSs were higher in cats from single compared with multi-cat households. CSSs were lower for cats that showed a more ‘pessimistic’ response in the JBT, suggesting these cats appeared to be less stressed. JBT results did not vary depending on the presence of, or reports of agonistic behaviours between, cohabiting cats. These data suggest that mood states (as measured by the JBT) were not impacted by the social groupings investigated, and that cats from single-cat households showed more signs of stress (as measured by CSS) than those in multi-cat households. Alternative explanations cannot be discounted, particularly due to the narrow sample population and broad scope of husbandry conditions that were unaccounted for. Further research is warranted to explore the extent to which variables that could not be controlled may have confounded findings.

**Abstract:**

Although agonistic interactions between cats are often regarded clinically as a source of stress, there is currently limited research evidence regarding the welfare impact of keeping multiple cats as pets. The aim of this study was to compare welfare indicators between cats living in domestic single and multi-cat households, as well as between multi-cat households where agonistic behaviour was/was not reported by owners. Indicators included a spatial judgment bias task (JBT), where longer latencies to ambiguous probes are interpreted as being related to a more ‘pessimistic’ mood state, and the cat stress score (CSS), where high scores are indicative of high stress levels. Of 128 focal cats between the ages of 9–22 months, 94 were from multi-cat households, 126 had useable CSS data and 42 had JBT results suitable for analysis. CSSs were significantly lower for cats showing a more ‘pessimistic’ response in the JBT. It is possible that the cats that appeared to be the most relaxed may have been showing inactivity relating to negative affective states and/or were the least active/food motivated, and therefore slower in the JBT. CSSs were significantly higher in cats from single compared with multi-cat households, and did not vary with reports of agonistic interactions in multi-cat households. JBT results did not vary depending on the presence of, or reports of agonistic behaviours between, cohabiting cats. These data suggest that cats from single-cat households may be more likely to show signs of acute stress than those in multi-cat households. Alternative explanations are possible. For example, lower CSSs in the multi-cat group may reflect ‘relief’ effects resulting from separating cats for the test period, or inactivity relating to negative affective states. Due to the narrow sample population and broad scope of husbandry conditions, the potential for confounding variables limits the degree by which results can be used to inform causation of the relationships identified. Further research is warranted to replicate this work and explore potential confounders.

## 1. Introduction

Domestic cats are a popular companion animal, with approximately 10 million cats estimated to be kept in the UK as pets [1]. Although cats may appear to be well adapted to fitting in alongside human lifestyles, there are some aspects of modern living which may be a welfare concern. In particular, those working in clinical behaviour commonly report cases where cats adapt poorly to living in close proximity with other cats, especially those who are unrelated and/or unfamiliar [2]. With an estimated 43% of owned cats in the UK being reported as living with at least one other cat, and approximately half of these cats being reported to endure conflicts with cohabiting cats, this is an area of cat behaviour that may have a considerable impact on the welfare of owned cats [1,3].

The ancestral species of the domestic cat, the African Wild cat (Felis silvestris lybica) [4], was likely to have been largely asocial [5]. Despite this, domestic cats living in feral colonies can form cohesive social groups, typically consisting of a core group of related females which may be associated with several roaming males, where resource availability and distribution allows [6]. However, commonly occurring social groupings in domestic environments, such as the co-habitation of cats introduced as adults, and behaviours observed between co-habiting cats, are considerably different from those in naturally occurring groups [7,8]. Close contact between incompatible cats has been suggested to be an important cause of undesirable stress-related behaviours [2,9]. Behaviours that owners find problematic can have further impact on welfare through consequences, such as relinquishment to shelters [10] or euthanasia. Given the apparent variation in social compatibility between cats, evaluating the degree to which contact with conspecifics may be perceived as positive or negative to individuals is an important area of research.

Measuring the welfare of domestic cats is a growing area of research that has thus far predominately been applied to controlled environments such as shelters or research facilities [11]. Methods of welfare assessment for cats in different social environments have mainly involved changes in physiological and behavioural parameters during or after a perceived challenge. Examples include activation of the hypothalamic pituitary–adrenal stress response pathway [12,13,14], as well as visual behavioural responses to stress, measured using an integrated behavioural observation measure for stress assessment, the ‘cat stress score’ (CSS) [15,16,17,18,19,20]. Prospective longitudinal data have also been used to examine the differences between multi/single-cat households [21]. The results of previous studies have yielded conflicting results which, despite highlighting a number of potentially important factors, have yet to provide clear evidence for the welfare implications of cat sociality [22].

One limitation of the current welfare assessment tools developed for use in cats is the difficulty in identifying the valence (positive or negative) of the animal’s emotional state [11]. Judgment biases have been used to evaluate welfare state based on the valence of emotions, since animals experiencing putatively poor welfare, such as being housed in unpredictable environments or frequently being exposed to ‘unpleasant’ experiences, have a more ‘pessimistic’ reaction to ambiguous stimuli in judgment bias tests (JBT) [23,24,25]. This welfare measure appears to be sensitive to the effects of social stress [26]. JBTs have been used in a range of species, and we have adapted protocols in order to develop a spatial JBT suitable for application to pet cats in the home environment [27,28,29].

CSSs [30] were used in this study as an additional welfare indicator and for comparison with JBT results. CSS scales were adapted with the addition of further categories [31] to increase sensitivity.

The objectives were to:

1. Investigate whether measures of relative judgment bias co-vary with CSSs. The CSS is believed to measure discrete behavioural responses to recent stress exposure, whereas judgment bias varies with underlying (and hence potentially longer term) affective states [32]. Hence, we would not expect to find a direct correlation between these measures.
2. Assess the welfare of pet cats based on the presence of, and relationships with, other cats in the household, using the JBT and CSS.
  - Cats in multi-cat households were hypothesized to show a longer latency to one or more ambiguous probes in a JBT (i.e., show a more ‘pessimistic’ judgment bias) and have higher CSSs compared to those living in single-cat households.
  - Cats from multi-cat households where the focal cat was reported to show and/or be the recipient of agonistic behaviour were hypothesized to show a longer latency to one or more ambiguous probes in a JBT and higher CSS compared to those who did not exhibit/were not the recipients of agonistic behaviour.

## 2. Materials and Methods

### 2.1. Study Population

The study population comprised 241 domestic pet cats from 105 different households. Of these, 128 cats were ‘focal’ cats, registered with two existing UK longitudinal questionnaire-based studies with the University of Bristol—the ‘Bristol Cats’ project and ‘Cats Longitudinal Analysis of Welfare Study’ (C.L.A.W.S). Focal cats were between 9–22 months of age at the time of data collection. This age range was selected due to restrictions on the availability of cats in the existing cohorts. The remaining cats in the study population were all of those co-habiting with the focal cats. The sample for this project was recruited from Southern England, the Midlands and South Wales. Owners were recruited via email, or telephone contact if they did not have a registered email address/had indicated a preference. Visits were carried out between 21 October 2013 and 26 May 2014. All owners gave fully informed, written consent to take part in the study. 

The cats were pets, kept primarily for the purpose of owner companionship, living in the owner’s homes. The majority of focal cats had both indoor and outdoor access (81.3%), a proportion of which remained stable across the multi-cat groups (81.3%) and agonistic behaviour groups (78.7%). Other husbandry conditions varied between cats and households, although several aspects of husbandry were recorded, these variables were not factored into statistical analysis due to the restricted sample size and broad scope of variation that existed. Potentially confounding variation that existed between the selected groups should therefore be considered a limitation of the study. Due to the nature of the study population (pet cats owned by participants who were volunteering their time to take part), the only experimental control placed on the cats outside of testing was to ask owners to avoid feeding focal cats for at least three hours prior to the arranged visit times in order to attempt to control for the effects of satiety. Based on the results of piloting [29], it was anticipated that roughly half of focal cats would complete the JBT, so attempts were made to recruit twice the desired number. Focal cats were excluded from the study if they were pregnant or lactating, on any pharmacological therapy likely to affect behaviour, were currently undergoing behaviour therapy, had any current medical illness, had a history of infectious disease with potential carrier status or were known by their owners to be fearful of strangers or handling.

The average age of focal cats was 14.46 months; 61.7% were male, of which two cats were not neutered. The remaining cats (38.3%) were neutered females. The proportion of males was slightly lower but stable across the multi-cat groups (47.7%) and agonistic behaviour groups (48.9%) examined. 

A total of 94 (73.4%) focal cats lived in multi-cat households, and the total number of cats in these households ranged from 2–8 (see Table 1 for frequencies). Information regarding the focal cat’s agonistic interactions with other cats in the household was collected via a verbal questionnaire, with the exception of one eight-cat household where the cats were kept separated and so no data regarding agonistic interactions were collected. CSS and JBT data were only collected for focal cats. Where households contained more than one cat registered to the ‘Bristol Cats’ or ‘C.L.A.W.S.’ studies, aged between 9–22 months, multiple focal cats from the same household were tested. No more than two focal cats were used from any one household, and two cats were selected at random from eligible cats using a random number generator [33]. The number and percentages of focal cats used for analysis of CSS and JBT data are shown in Table 1.

### 2.2. Experimental Protocol

Cat owners were visited in their homes for data collection. All data collection was conducted in one room of the house—ideally a quiet room free from distractions, with adequate space for the JBT. At least one owner would usually be present for the entire process, in addition to the lead researcher (researcher A). An additional researcher (researcher B) was present for the initial visit and, in some cases, subsequent visits. Researcher B was not always the same person.

The chronological stages of the experimental protocol are described below. The average time from arrival to the subject’s home to the time of completion for one cat on the initial visit was 45 min, and 30 min for each subsequent visit. All equipment was wiped down with Dettol^®^ antibacterial spray between visits. Each focal cat was visited on up to five separate occasions to complete training and testing for the JBT.

#### 2.2.1. Habituation 

Habituation times were based on those described by Kessler and Turner [30] and tested during the piloting stage of this project [29]. This was 10 min on the first visit and five minutes for each subsequent visit. Habituation began once the researchers, owners and cat were in the room of the house selected for data collection, and the JBT equipment (excluding the food bowl) was placed out for the cat to investigate. 

#### 2.2.2. Verbal Questionnaire (Multi-Cat Households Only) 

At the initial visit, owners of multiple cats read a list of social behaviours that had previously been described as agonistic/indicative of cats that do not perceive one another as part of the same social group [2,34]. They were asked to report whether they had witnessed the focal cat show, or were a recipient of, any of these behaviours over the last month, with any other household cat. The behaviours, as described to owners, and their abbreviated definitions, are as follows:

- Fluffing: ‘fluffing up’ at one another (when the hairs go on end making the cat look bigger).
- Freezing: freezing and staring at one another—for example, in corridors or doorways, for five seconds or more at a time.
- Vocalising: hissing/spitting/yowling/growling at one another.
- Blocking: blocking or inhibiting one another’s movements or being reluctant to pass one another in tight spaces (e.g., corridors and doorways).
- Aggression: fighting (aggression not occurring out of play), e.g., scratching/biting, chasing and attacking.

#### 2.2.3. CSS

The adapted 9-point CSS [31] was used. This scoring system added half points to the original scale [30]. In the current study, the same scale points were used but were named as complete integers; hence, a score of 2.5 became a score of 3, and score 3.5 became a score of 4 [31]. This resulted in a scale ranging from 1 = fully relaxed to 9 = terrorized. Scoring was conducted by selecting the score that best fitted with the majority of behavioural signals shown by each cat. Where behavioural signs were ambiguous between points on the scale, the lowest possible score was given.

The scores for each cat were taken immediately after the habituation period at every visit, meaning that multiple scores were collected for cats undergoing JBT training and testing. Three scores were taken at each visit, with a two-minute interval between each. Video footage was taken for the duration of scoring in order to assess reliability by comparison, with scores given by an independent observer. The mean CSS across all visits for each cat was used in data analysis.

#### 2.2.4. Feline Temperament Profiling

At the initial visit only, following assignment of CSSs, an adapted version of the Feline Temperament Profiling (FTP) scoring system [35] was used to assess the cats’ behavioural responses to the researcher and screen out those unsuitable for further testing. The protocol for FTP was adapted to enable rapid assessment of whether cats were showing an ‘unacceptable’ level of fear, which would suggest they would not respond well to the level of handling required for JBT (and therefore ensured their welfare was protected). An ‘unacceptable’ level of fear was reached if the cat showed three or more ‘potential’ indicators (e.g., dilated pupils, avoiding eye contact) or any one clearly fearful reaction (e.g., aggression or hiding). If this was the case, the assessment was stopped, and the judgment bias training was not attempted. Steps 7–10 of the FTP protocol were omitted, as they were deemed unnecessary for the purposes of this study. A copy of the adapted protocol used can be found in the supplementary information.

#### 2.2.5. JBT

A spatial JBT was used, which consisted of a training phase followed by a test phase. During the training phase, cats were presented with a bowl which was either baited with a treat on one side of the experimental set up, or a bowl which was empty on the opposite side of the experimental set up. Training was completed once the cat had reached criteria to demonstrate that they were more likely to anticipate a food reward in the baited bowl location compared with the empty bowl location. During the following test phase, ‘probe’ bowls were presented at intermediate locations, and the cat’s latency to approach each bowl was recorded as an indication of judgment bias. The detailed protocol is described in full below, based on that of Tami et al. [28] and a similar protocol developed for dogs [27]. This protocol has been piloted for use in pet cats in the home environment [29], and the results were used to inform the methodology of this study. Adaptations resulting from piloting included: the use of a door that opens from the centre to reduce the risk of side biases developing during training; the use of a spatial measure in order to determine whether cats had ‘checked’ bowls instead of relying on observer perception; the use of a novel food bowl instead of the cat’s own bowl to avoid variation in bowl height/size and prior associations; and recruiting the owner’s help to restrain the cat instead of a secondary researcher, where possible, to reduce potential stress caused by handling by an unfamiliar person.

Judgment bias training started at the initial visit, following FTP (excluding those cats scored as having an ‘unacceptable’ level of fear). The JBT could take up to five sessions for the cat to learn and complete, and it was always carried out after the habituation period and CSS observations.

A minimum of two people were required to be present for each training session and testing. Researcher A was always the same person throughout data collection, and was responsible for placing the bowl in position and measuring latencies. The owner would usually fulfil the role of the second person, handling and releasing the cat. A second researcher (B) was present on the first day of training to help the owner. If the owner was physically unable to handle and release the cat themselves, researcher B would attend subsequent visits to fulfil this role (*n* = 7).

The study received human and animal participant ethical approval from the local University ethics committee.

##### Training Phase

The training phase was made up of a maximum of 60 trials, with 15 trials per session. Cats had one training session per day, and training sessions were conducted on consecutive days. In some cases, this schedule had to be adapted to fit with owner availability. One cat had two training sessions on the same day, and 13 cats did not have training on consecutive days.

**Figure 1:**
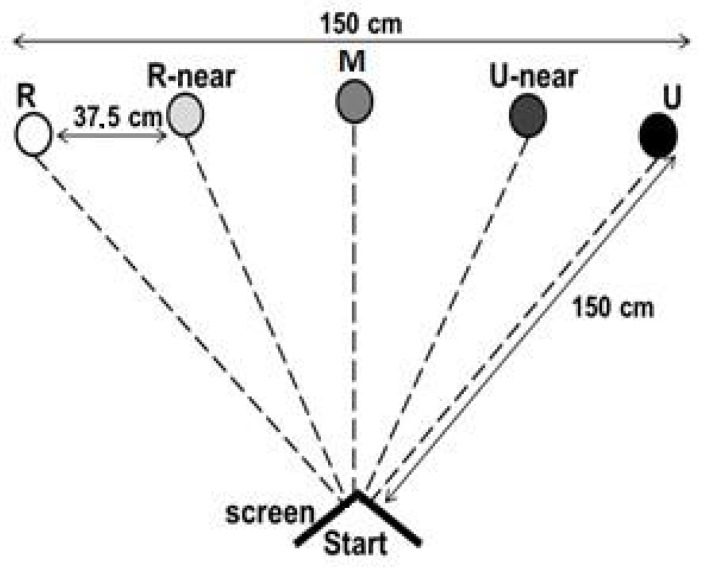
Experimental set up for judgment bias test.

The apparatus was set up as shown in Figure 1, using masking tape to mark bowl locations on the floor. A clear laminate plastic semicircle measuring 60 cm across was placed underneath the bowl every time it was presented, at all locations.

The positions of the rewarded (R) and unrewarded (U) bowl locations remained constant for each cat, but were randomized between cats using an online random number generator [33].

The cat was held behind the screen by the owner (or researcher B) while the bowl (which was novel to the cat) was baited/not-baited with a food reward (one beef Whiskas^®^ Temptations biscuit; this was kept standard across all cats to prevent variation in responses due to food type).

The bowl was always baited in the same way; the bowl was picked up by researcher A, taken to the baiting location (halfway between the two bowls) and either baited or not baited. Either way, the box with the food in it was opened and closed, and a treat was placed in the bowl (then removed if it was a non-baited trial). The bowl was then tapped twice with a pen to earn the cat’s attention and signal that a trial was about to begin. 

The bowl was placed in either the ‘R’ or ‘U’ position, on top of the plastic semicircle. The order of ‘R’ and ‘U’ trials was pseudo-random, with no more than two in the same location continuously to decrease the likelihood of side biases developing. 

The barrier (two solid panels that opened outwards, creating a ‘doorway’ in the centre for the cat to proceed through) was then opened by the owner or researcher B, ensuring minimal distraction and reducing the risk of side bias. 

The cat was released as soon as the barrier was opened; facing straight forward, the time taken for the cat to move from the starting point and place a foot onto the plastic semicircle was recorded.

The cat was given up to 45 s to reach the semicircle; if the cat took longer than this, the maximum time of 45 s was recorded. 

Once the cat had reached the semicircle, or 45 s had elapsed, the cat was recalled/moved back behind the barrier by the owner or researcher B. If the bowl was baited, the cat ate the treat before being recalled. 

At the first training session, the cat was presented with three ‘R’ and three ‘U’ trials in a pseudo-random order (as described above). During these trials, researcher A gave two additional taps on the edge of the bowl with a pen, up to three times per trial, to earn the cat’s attention and aid learning. 

In all subsequent training sessions, ‘R’ and ‘U’ trials were presented in a pseudorandom order and no additional taps were provided. 

The cat was said to have learned the task when it was consistently visiting the ‘R’ location faster than the ‘U’ location. Consistently faster was defined as: for any six consecutive trials in one discrete training session, the longest latency to reach the rewarded side must be at least half a second less than the shortest latency to reach the unrewarded side. 

Each cat was given a maximum of 60 learning trials to reach the criterion. Once the cat had learned the task it was presented with the test phase. 

##### Test Phase

The test phase consisted of 14 trials. This was ideally run the day after the cat reached the criterion; however, in 18 cases, this was not possible due to the need to fit in with the time schedules of owners (range = 1–9 days). Although this variability was unavoidable, it may have impacted on learning the discrimination as highlighted in the discussion. 

During the test phase, the cat was presented with bowls in intermediate locations (‘probes’), as shown in Figure 1. The bowl nearest the rewarded location was termed ‘R-near’ (nearer rewarded), the bowl nearest the unrewarded location was termed ‘U-near’ (nearer unrewarded) and the central bowl was ‘M’ (middle). These positions were equidistant from the ‘R’ and ‘U’ locations and were not reinforced with a food reward. 

The probes were presented in a pseudo-random order, interspersed with the reference ‘U’ and ‘R’ locations. For the final trial the bowl was placed in the ‘U’ location and baited to ensure the cat was learning spatial associations and not following olfactory cues (the false negative, ‘U = R’). Each probe location was only presented once in order to prevent potential confounding effects of a reduced latency to these positions through repeated presentation due to cats learning that these locations were unrewarded [27]. Baiting/non-baiting of bowls was performed in exactly the same way as the training trials. The cat’s latency to reach each probe was measured when the cat placed a paw on the plastic semi-circle surrounding the bowl. The precise bowl locations and order of occurrence for the test phase are presented in the Supplementary Materials. 

### 2.3. Statistical Analysis

In order to account for potential bias caused by testing multiple focal cats from the same household (*n* = 23), all statistical analysis presented in this section has been replicated with a reduced sample size (*n* = 105) to eliminate repeated testing of cohabiting cats. An online random number generator was used to select a single focal cat from each household to include in this analysis [33]. This resulted in a reduced population of 103 focal cats with useable CSS data, and 35 with useable JBT data. In this reduced population, statistical significance and the direction of the relationships identified were unaffected, demonstrating a low likelihood of bias caused by testing multiple focal cats from the same household. Due to the already limited sample size, results from the full population of 128 focal cats are presented and discussed below. Dependent variables were skewed and could not be normalised using log or square root transformations; hence, non-parametric methods were used. Data were analysed using IBM SPSS version 21 unless otherwise stated. 

#### 2.3.1. CSS Preliminary Analysis 

To ensure consistency in scoring CSS, the lead researcher and an independent scorer assessed sets of 19 videos repeatedly in order to ensure their methods were reliable; this was before they scored the study population. A ‘strong’ level of agreement between scores after two repetitions was achieved (weighted Kappa = 0.768). Inter-rater reliability was ‘moderate’, and was assessed by comparing 20 scores taken during visits with scores given using the video footage of these visits by the independent scorer (weighted Kappa = 0.531). Weighted Kappa results were assessed, and interpretation was determined using the ‘StatsToDo’ online resource [36]. 

Although the CSS is often described as a scale, differences between integers are not necessarily equivalent; hence, they were analysed as categorical variables. Categories were combined into three groups (CS1–2; CSS 3; CSS 4–6) because of the low number of cats showing very low or high CSS values.

#### 2.3.2. JBT Analysis

Wilcoxon signed ranks tests (*T*) were used to compare latencies to each bowl location presented during the JBT. Kruskal–Wallis tests were used to compare categories of CSS with probe latencies in the JBT.

#### 2.3.3. Comparing Welfare Measures Based on Social Conditions

Mann–Whitney U tests (*U*) were used to compare the distribution of probe latencies for cats that lived in multi-cat households with those from single-cat households, as well as for cats that lived in multi-cat households where there were agonistic inter-cat interactions and those where there were not. Chi-squared tests (χ^2^) were used to compare CSS groups between these household types.

## 3. Results

### 3.1. CSS

**Figure 2:**
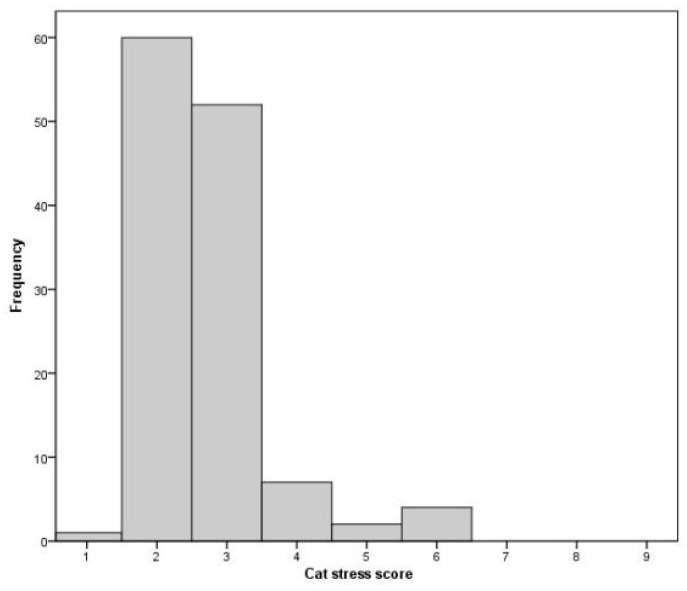
Histogram showing the frequency distribution of the average cat stress score for each cat with useable CSS data, taken from three measures per visit (*n* = 126).

CSS data were not collected for two focal cats, as they remained hidden during visits. For the remaining cats (*n* = 126), the average CSS for each cat over all visits was used in the analysis (Figure 2). The median score was three, with an interquartile range of one.

### 3.2. JBT 

A total of 42 (32.81%) focal cats had JBT results that were used in analyses. A further 12 cats completed the JBT but had either travelled on average faster to the U compared with the R position during the test phase or had become uninterested/distracted in the test phase. The remaining 76 cats did not reach the JBT learning criterion; of these, 30 cats showed behavioural signs indicative of fear or frustration; 17 were not interested in the food reward; 13 did not pass FTP; 11 lost interest in the task; two showed a combination of the above reasons; and one became injured between training sessions (due to an unrelated incident).

**Figure 3:**
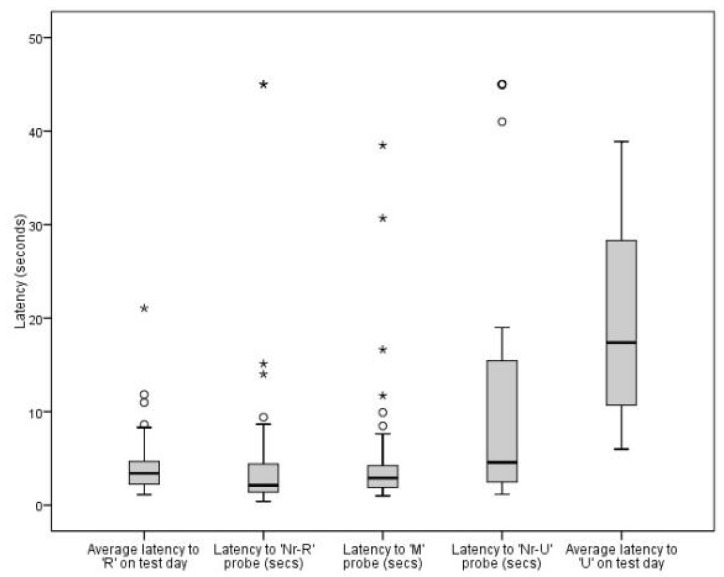
Box plot showing latencies to bowl locations during judgment bias testing for 42 cats with useable data. Outliers (^◯^) and extreme outliers (*) are identified.

The number of trials taken to reach the learning criterion for the JBT was positively skewed. The median number of trials was 14.5, ranging from the minimum number possible (6) to 51 trials. The interquartile range was 13. Initial exploration of the latencies to ambiguous probes for those cats that completed the JBT showed similar results for latencies to the R, R-near, M and U-near bowl locations (Figure 3). Notably, there was a high degree of variation between individuals in the population, with a proportion of cats having much longer latencies to all locations.

Median latencies to the U location were significantly longer than to all other locations on test day: R (*T* = 902.0, *p* = <0.001), Nr-R (*T* = 3.0, *p* = <0.001), M (*T* = 23.0, *p* = <0.001), Nr-U (*T* = 165.0, *p* = 0.003 and the ‘false negative’ U = R (*T* = 486.0, *p* = 0.005). These results confirmed that the cats who had reached the learning criterion could distinguish between the reference locations, and perceived this location as least likely to be rewarding. This also demonstrates that the cats were unlikely to respond to olfactory (or other) cues to locate the treats, and that their response was based on prior learning about the location. 

Using a Bonferroni corrected *p* value (0.008), median latencies to the R location on test day were not significantly different from latencies to the Nr-R (*T* = 453.0, *p* = 0.563) or M (*T* = 521.5, *p* = 0.029) probes. This suggests that cats did not perceive these intermediate probes as less likely to be rewarding. Median latencies to the R location were significantly shorter than latencies to the Nr-U probe (*T* = 776.5, *p* = <0.001), suggesting that cats were less likely to expect the bowl to contain a reward in the Nr-U position. 

When comparing intermediate probes with one another, latencies to the M probe were not significantly different from latencies to the Nr-R probe (*T* = 282.0, *p* = 0.085), suggesting that cats did not differentiate between the two in terms of reward probability. Latencies to the Nr-U probe were significantly longer compared with latencies to both the Nr-R (*T* = 765.0, *p* = < 0.001) and M (*T* = 698.5, *p* = 0.002) probes, supporting the conclusion that cats perceived this location as less likely to be rewarding. 

### 3.3. Comparing CSS and JBT Data

**Figure 4:**
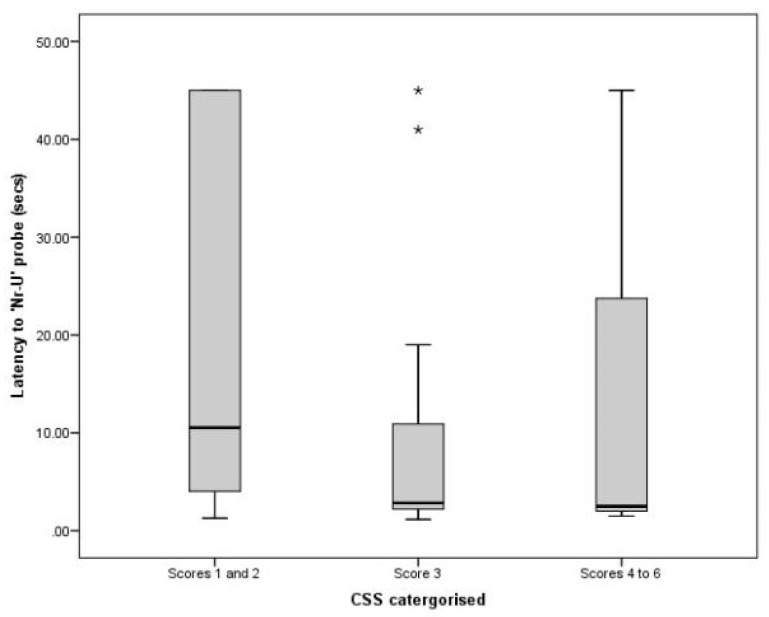
Box plot showing variation in latency to the near-unrewarded probe between different cat stress score categories. Extreme outliers (*) are identified.

**Figure 5:**
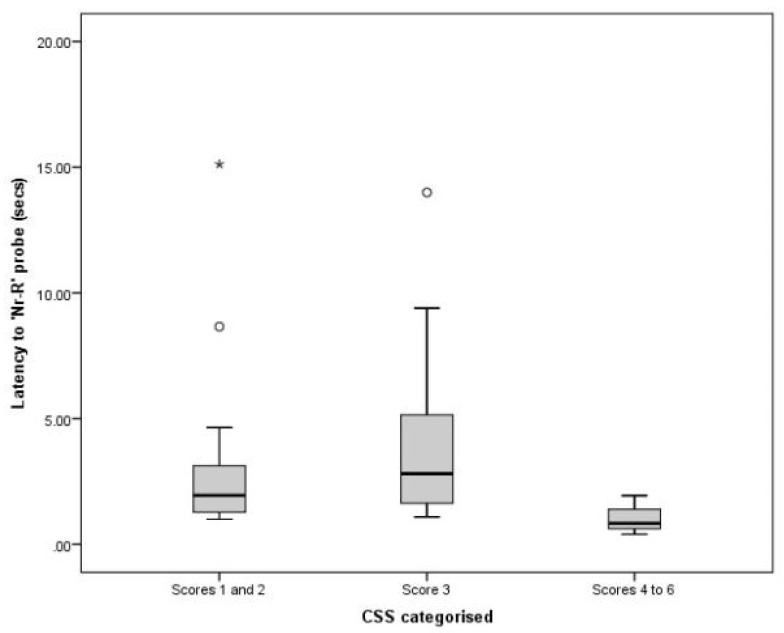
Box plot showing variation in latency to the near-rewarded probe between different cat stress score categories. Outliers (^◯^) and extreme outliers (*) are identified.

Categories of CSSs were significantly associated with latency to the Nr-U probe (χ^2^ = 6.07, df = 2.0, *p* = 0.048; Figure 4), and showed a trend towards association with the Nr-R probe (χ^2^ = 5.90, df = 2.0, *p* = 0.052; Figure 5). No significant association was identified between CSSs and latency to the M probe (χ^2^ = 0.30, df = 2.0, *p* = 0.861). Post hoc analyses suggested a significant difference between scores 1–2 and a score of 3 (*U* = 101, *p* = 0.012) for Nr-U probe latency, where cats with the lowest stress scores were slower to reach the Nr-U probe. For the Nr-R probe, cats with CSSs of three appeared slower to reach the bowl compared with those cats that had the highest CSSs of 4–6 (*U* = 7, *p* = 0.031). This trend was the only result that was not replicated when statistical analysis was repeated with a smaller sample size of 105 cats from separate households. This was due to cats scoring 4–6 with useable JBT data being randomly removed from the study population. 

### 3.4. Comparing Welfare Measures between Multi-Cat and Single-Cat Households

Of the 42 cats that fulfilled the inclusion criteria for analysis of JBT data, 33 (78.5%) were from multi-cat households. Probe latencies did not differ significantly between single and multi-cat households (Nr-R: *U* = 134.0, *p* = 0.673; M: *U* = 101.0, *p* = 0.152; Nr-U: *U* = 122.5, *p* = 0.432). Effect sizes were calculated to further examine differences between groups because of the small sample of cats in single-cat households. In all cases, the estimated effect size was small (<0.3).

**Figure 6:**
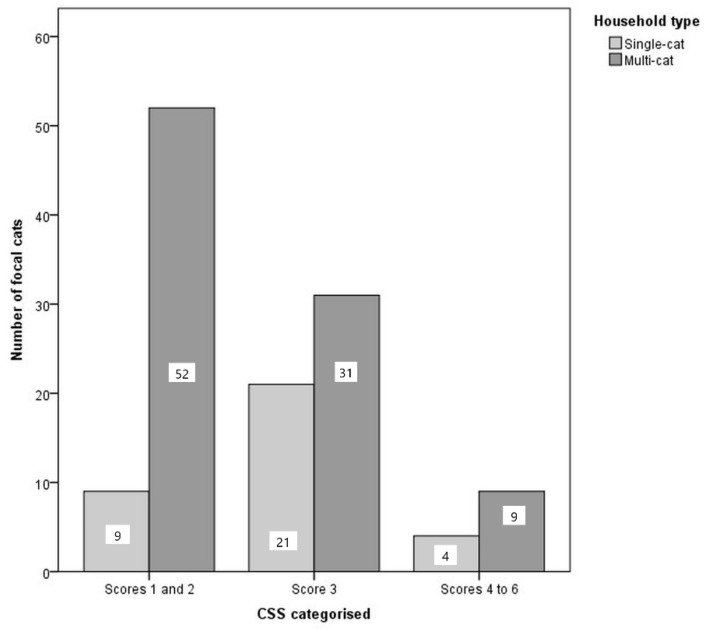
Clustered bar chart showing the distribution of cat stress scores from cats in single-cat households and multi-cat household (*n* = 34 single cats, 94 multi-cats).

A significant difference was identified between CSSs in multi-cat households compared to single-cat households (χ^2^ = 9.465, *p* = 0.009, *n* =126 cats with CSS data). Post hoc analysis using a Bonferroni corrected *p* value (0.017) indicated that cats from multi-cat households were more likely than solitary cats to have CSSs of 1–2 (χ^2^ = 8.977, *p* = 0.003, *n* = 126), and solitary-cat households were more likely than multi-cats to have CSSs of 3 (χ^2^ = 8.977, *p* = 0.003, *n* = 126). No significant relationship was identified between household type and CSSs of 4–6. Figure 6.

### 3.5. Comparing Welfare Measures Based on the Occurrence of Agonistic Interactions in Multi-Cat Households

One of the 94 focal cats living in multi-cat households was kept separately from the other cats in the home, and therefore excluded from analysis. A total of 70 of the 93 remaining cats displayed and/or were recipients of agonistic behaviour (fluffing, blocking, freezing, aggression and/or vocalising) from other cats in the home (75.3%). CSS data was collected for 68 of these cats. CSS categories did not vary significantly between cats who displayed/received agonistic behaviour and those who did not (χ^2^ = 4.33, *p* = 0.115, *n* = 92).

Of the 33 cats from multi-cat households that completed the JBT, 26 (78.8%) displayed and/or were recipients of agonistic behaviour from other cats in the home. Probe latencies did not differ significantly between these cats and those that did not show/were not recipients of agonistic behaviour (Nr-R: *U* = 87.0, *p* = 0.880; M: *U* = 60.0, *p* = 0.183; Nr-U: *U* = 86.0, *p* = 0.846). Effect sizes were calculated to further examine differences between groups because of the small sample of cats in non-agonistic households. In all cases, the estimated effect size was small (<0.3).

## 4. Discussion

The measures used to investigate the welfare implications of cat housing conditions in this study included the CSS and the JBT. These measures were first compared with one another to investigate the extent to which they co-varied, and then compared between groups of cats in order to assess welfare in relation to different social housing conditions.

The broad scope of variation that may have existed between the social groups examined in this study could have potentially confounded results (for example, the degree of relatedness between cats, the age at which they were introduced, relationships with neighbouring cats or with other household pets were not recorded or taken into account). A potentially confounding variation that existed between the selected groups should therefore be considered a limitation of the study, and one which limits the extent to which results can be used to inform causation.

### 4.1. Usability of CSS and JBT Welfare Measures in Home Environments

The CSS is an observational measure of stress which is most likely to reflect a cat’s response to its immediate environment at the time of testing. The CSS was a quick, easy and non-invasive measure to take in the homes of pet cats. A moderate-high level of within and between-scorer reliability was achieved for CSS data. This can be considered a limitation of the study, and one which may have been improved by increasing the number of training sessions scorers completed. 

CSSs within the population had a relatively narrow distribution, with the vast majority of cats scoring either 2 or 3, despite the use of an ‘adapted’ scale with additional points [31]. This is likely to be because the majority of cats were relaxed in their home environment and were given time to habituate to the researchers and equipment. Further increases in the sensitivity of the CSS to pick up on differences between cats with low or intermediate scores may be a beneficial area of future research when assessing cats in their home environment. 

An important consideration related to the CSS is the difficulty in attributing scores to emotional valance, especially ‘mid-range’ scores. Low scores of 1–2 reflect behavioural signs of relaxed cats, and scores near the mid-range (3–5) indicate more alert cats; however, the behavioural ‘stress’ signals relate more to general activity (and are not specific to negative emotions). Scores of six and upwards indicate behavioural responses to stress that are more likely to be associated with negative emotional states. No cats in this study had scores of seven or over. The higher scores identified in this study may therefore simply reflect cats that were showing signs of being more alert/active, and not in distress. It is unlikely that cats with very low CSSs in this study were showing acute stress-related inactivity [37], as these cats were screened out as unsuitable for testing. It is, however, possible that inactivity associated with low CSSs may have been associated with a ‘depression’-like state thought to be associated with negative affective states [38].

Unlike the CSS, the JBT aimed to gain a measure of longer-term affective states. Under one-third of the original sample of focal cats had JBT data suitable for analysis, a similar proportion reported in previous studies applying the JBT to cat populations [25,28,29]. The most common reasons for incompletion of the task were behavioural signs indicative of fear, or disinterest in the food reward. This is likely to have biased the sample of cats with useable JBT data to those that were less fearful and more food motivated. Developing ways to reduce the number of cats being excluded from JBT due to fearful responses will be important for future work. This may involve reducing the potentially fear-inducing elements of the JBT protocol (e.g., handling/restraint) as much as possible, and altering the habituation protocol. It should also be considered that, although food is likely to be the easiest reward to manipulate for the purposes of the JBT, the preference that cats may have for social interaction with people could have influenced results and reduced the ‘success rate’ of training [39].

Conducting the JBT in the owners’ homes placed potentially confounding factors out of experimental control. It was necessary to fit testing around owners’ schedules, meaning that, in some cases, testing did not occur the day following a cat reaching training criterion. The high variability in environmental conditions and pre-existing associations cats had about the environment may have influenced their performance of the task. Household distractions are also likely to have affected results. Probe latencies in the JBT were especially sensitive to distractions, and to the fact that each intermediate was only presented once to prevent learnt reduction in responses to repeated presentations [40]. Although six cats were excluded from analysis as a result of distraction, it is possible that others also became distracted but that this was not observed during the trials. Relying on owners to restrain the cats during testing resulted in differences in handling ability, which could have affected the likelihood of cats completing testing and may have influenced their affective state. The presence of unfamiliar researchers may have had a similar affect. Food motivation varied between cats and would be influenced by husbandry systems outside the control of the study, which is likely to have affected performance in the JBT, despite attempts to control for satiety by instructing owners not to feed their cats for 3 h before visits. 

### 4.2. Comparing CSS and JBT Results

Cats with CSSs of 1–2 (relaxed or very relaxed) had longer latencies to reach the near unrewarded probe in the JBT compared to those with CSSs of three (alert). Similarly, cats with CSSs of three showed a trend for longer latencies to the Nr-R probe compared with those cats with highest CSSs of 4–6. 

These were unexpected results that indicated low CSSs were associated with a more ‘pessimistic’ judgment bias. It is possible that the low stress scores identified in more ‘pessimistic’ cats may be a reflection of ‘relief’ resulting from separation from other cats or other longer-term stress causing factors that had not been recorded in this study, but which may have resulted in a negative judgment bias. 

The latency to each bowl is influenced by the relative motivation of cats to achieve the food reward, and cats that are not alert and/or interested in the task may be both more relaxed in appearance and slower or less interested in investigating novel locations if there is a chance of non-reward. It is possible that this relative inactivity associated with low CSSs was caused by a ‘depression’-like state associated with negative affective states [38].

### 4.3. Comparing Groups of Cats Using JBT and CSS Welfare Measures 

Cats from multi-cat households were more likely to have ‘relaxed’ or ‘very relaxed’ CSSs of 1–2 compared with those from single-cat households; the latter were more likely to have an ‘alert’ score of 3. There was no difference between groups for the highest CSSs (4–6). CSSs did not vary between multi-cat households depending on the occurrence of reported inter-cat agonistic behaviours. 

This result was contrary to our hypothesis and indicates that cats from single-cat households were more ‘alert’ due to stress. Young cats living in groups from domestic settings have previously been identified as less stressed than their single-housed counterparts, and so this finding may be a valid representation of stress, particularly due to the young age of the focal cats [14]. Again, there are other possible explanations. For example, cats from multi-cat households were typically separated from other cats for at least five minutes prior to the recording of stress scores, and they may have been showing enhanced relaxed behaviours as positive emotional responses to this separation and the opportunity for more one-to-one time with their owners or researchers. It is also possible that the low CSSs identified for multi-cats in this study were reflective of negative affective states [38].

No significant differences were identified for JBT results when comparing single-cat households with multi-cat households, or when comparing multi-cat households based on the occurrence of agonistic interactions. These results may indicate that the presence of other household cats or the occurrence of agonistic behaviours between household cats do not have a significant impact on affective state. This may be because positive effects of living with other cats counteract the negative impact of agonistic encounters. It is also possible that the JBT may not be sensitive enough to detect changes in affective states caused by chronic social stress due to a ‘masking’ effect that other, more transient, changes in the environment may have [41].

It is possible that the lack of significant JBT results in respect to the social conditions of cats measured in this study can be attributed to the test used being inaccurate in recording the underlying affective state of participating cats. This explanation is deemed likely because JBT latencies recorded were similar between the R, Nr-R, and M locations. When significant differences were found, they were detected at the Nr-U probe, which was least likely to be perceived as rewarded. The lack of distinction between probes could be a result of the U position not being punishing enough, due to the fact it is a lack of reward rather than an overt risk [42]. This explanation may be further supported by previous research comparing groups of cats using a similar JBT set up, which did not identify differences between the groups tested.

It is possible that the method for categorising groups of household cats into ‘agnostic’ or ‘non-agonistic’ did not successfully capture differences in social groupings that have the most significant effect on cat welfare. Agonistic behaviours were owner reported due to restraints on time, controllability of the cats’ movements and the level of disturbance caused to owners. This would have resulted in a higher risk of inaccuracies caused by interpretation or awareness. For example, owners may have struggled to differentiate between social play and agonistic aggression. Responses were reduced to a binary (occurring/not occurring) variable for analysis. Cats showing/receiving rare or transient signs of aggression would therefore be categorised in the ‘agonistic’ group alongside those with frequent and severe aggression. This method of categorisation may have masked differences between groups and resulted in limitations related to owner reports, including inaccuracies or misinterpretations. It is also possible that other social behaviours, such as the occurrence of positive interactions or avoidance behaviours, have a greater impact on the welfare of groups of cats than the presence of agonistic behaviours.

Lastly, the lack of significant results identified between the agonistic and non-agonistic groups may be interpreted as evidence that the JBT and CSS are not valid indicators of welfare in the home environment, as the occurrence of agonistic behaviours between cats can be interpreted as an additional indicator of poor welfare [2,21,43].

## 5. Conclusions

Despite limitations in methodology and demographics of the study population which limit interpretation of causation and extrapolation of results to the general population of pet cats, this study has demonstrated the potential use of a spatial JBT in the home environment. The results can be used to improve the efficacy of such testing protocols, should they be repeated in future research, including ensuring the environment used for testing is as ‘quiet’ as possible in order to prevent distraction from the task and reduce potentially confounding transient emotional states. 

CSSs were lower for cats that showed a more ‘pessimistic’ response to the Nr-U probe in the JBT, suggesting that ‘pessimistic’ cats appeared to be less stressed. It is possible that the cats who appeared to be the most relaxed were also the least active/food motivated, and therefore slower in the JBT than the more ‘alert’ cats. This relative inactivity may be reflective of negative affective states [38].

CSSs were higher in cats from single-cat households compared to multi-cat households. These results may indicate that young cats from multi-cat households are less stressed or, alternatively, that in the context of this study, low scores of 1–2 were potentially indicative of ‘relief’ responses or inactivity that may be related to negative affective states.

JBT results did not vary depending on the presence of, or reports of agonistic behaviours between, cohabiting cats. This suggests that aspects of social housing we measured may not have influenced the affective states of the cats involved. Other explanations, including questioning the validity of the JBT (and CSS) as an accurate measure of welfare in this study, have also been highlighted.

Factors that may have resulted in the high level of variation in JBT results include aspects of husbandry and social interactions, such as those with neighbouring cats, that were not under experimental control. The majority of the cats in this study had unsupervised access outdoors, introducing a high degree of variability and potentially stressful experiences.

Care should be taken when extrapolating the results of this study to the general population due to potential bias in the sample (cats of a specific age group belonging to owners who were already enrolled in existing cat epidemiology studies). Although prior screening of cats using FTP was important to ensure the welfare of cats included in the study, the removal of cats showing signs of fear prior to testing will have further biased the sample. The small sample size should also be considered a limitation when interpreting and attempting to generalise the results of this study. Despite this limitation, sample size calculations indicated that it was unlikely that significant relationships were missed.

## Figures and Tables

**Table 1:** The total study population, those with a useable cat stress score and judgment bias data.

Total Number of Cats in Household	Total Study Population		Study Population with Cat Stress Score Data		Study Population with Useable Judgment Bias Data	
	Number (%) of Households (Total = 105)	Number (%) of Focal Cats (Total = 128)	Number (%) of Households (Total = 103)	Number (%) of Focal Cats (Total = 126)	Number (%) of Households (Total = 37)	Number (%) of Focal Cats (Total = 42)
1	34 (32.4)	34 (26.6)	34 (33.0)	34 (27.0)	9 (24.3)	9 (21.4)
2	47 (44.8)	61 (47.7)	45 (43.7)	59 (46.8)	22 (59.5)	26 (61.9)
3	7 (6.7)	10 (7.8)	7 (6.8)	10 (7.9)	3 (8.1)	4 (9.5)
4	6 (5.7)	9 (7.0)	6 (5.8)	9 (7.1)	1 (2.7)	1 (2.4)
5	5 (4.8)	6 (4.7)	5 (4.9)	6 (4.8)	1 (2.7)	1 (2.4)
6	2 (1.9)	2 (1.6)	2 (1.9)	2 (1.6)	0 (0.0)	0 (0.0)
7	1 (1.0)	1 (0.8)	1 (1.0)	1 (0.8)	0 (0.0)	0 (0.0)
8	3 (2.9)	5 (3.9)	3 (2.9)	5 (4.0)	1 (2.7)	1 (2.4)

## Data Availability

The data presented in this study are available on request from cat-study@bristol.ac.uk. The data are not publicly available due to privacy restrictions.

## Supplementary Materials

The following are available online at https://www.mdpi.com/article/10.3390/ani11061793/s1, Adapted Feline Temperament Profiling protocol.
