# Supplementary material for: Cognitive and Composite Behavioural Welfare Assessments of Pet Cats between the Ages of 9–22 Months, Living in Single and Multi-Cat Households
Source: Animals (Basel). 2021 Jun 16;11(6):1793. doi: 10.3390/ani11061793 (PMC8234069; doi:10.3390/ani11061793)
Supplement: Supplementary file 1 [file animals-11-01793-s001.zip › animals-1230050-supplementary.pdf]

Bristol Cats C.L.A.W.S. University of BRISTOL

Assessment of fear of unfamiliar people

adapted from 'Feline Temperament Profiling' Seigford et al 2003

Following a 10 minute habituation period and 'Cat Stress Score' observations, begin to interact with the cat using the steps listed below. Indicate if the listed behaviours occur with a tick next to the relevant behaviour shown.

If you tick *three or more* behaviours listed for any step, or tick *any behaviour with a star (*)* on any step, this is defined as an 'unacceptable' level of anxiety/fear. Stop the assessment immediately; do not progress onto the next steps. The cat is unlikely to be confident enough to be used for judgment bias testing or puzzle feeders.

If three or more listed behaviours, or any starred behaviours are ticked on any step explain to the owner that we will not be coming back for subsequent visits. This is because we do not want to cause the cat any stress. Request video footage of the cat interacting with any other cats in the household, to be sent to us by the owner at a later date.

1. The tester should squat down about 5-6 feet away from the cat and call the cat several times. One hand should be extended. The cat:

	Tick all that apply
Avoids eye contact	
Watches with no approach	
Has large, dilated pupils	
Has its whiskers held back close to its face	
Swishes its tail quickly from one side to another	
Crouches on all four paws, with its body near to the ground	
Holds its head position low (on the plane of the body or lower)	
Retreats	
Hisses/Growls*	
Hides *	
Flattens ears *	

Other observations____

____

2. If the cat approaches the researcher during step 1, proceed to step 3. If the cat does not approach, move closer to the cat (about 3 feet away) and call again with hand extended. The cat:

	Tick all that apply
Avoids eye contact	
Watches with no approach	
Has large, dilated pupils	
Has its whiskers held back close to its face	
Swishes its tail quickly from one side to another	
Crouches on all four paws, with its body near to the ground	
Holds its head position low (on the plane of the body or lower)	
Retreats	
Hisses/Growls*	
Hides *	
Flattens ears *	

Other observations____

____

If the cat still does not approach after step 2, and shows any behaviour (including those that are not starred) listed in step 2 stop the assessment immediately and do not move on to the next steps. The cat is showing 'unacceptable' levels of fear/anxiety and is unlikely to be confident enough to be used in the Judgment Bias or Puzzle feeder tasks.

If the cat does not approach but shows no behaviours listed in chart two, proceed to approach the cat and move on to step 3.

3. After approaching or getting the cat to come, extend hand to cat while squatting (without making contact with the cat). Hand should be lower than cat's head. The cat:

	Tick all that apply
Avoids eye contact	
Holds its head position low (on the plane of the body or lower)	
Has large, dilated pupils	
Has its whiskers held back close to its face	
Swishes its tail quickly from one side to another	
Crouches on all four paws, with its body near to the ground	
Does not approach	
Retreats	
Hisses/Growls*	
Hides *	
Flattens ears *	
Strikes/attempts to strike hand – fast movement with claws outstretched *	
Bites/attempts to bite hand – fast movement, if the cat makes contact the bite is of fair force (enough to hurt or mark observer) *	

Other observations____

____

If the cat still does not approach after step 3, and shows any behaviour (including those that are not starred) listed in step 3 stop the assessment immediately and do not move on to the next steps. The cat is showing 'unacceptable' levels of fear/anxiety and is unlikely to be confident enough to be used in the Judgment Bias or Puzzle Feeder tasks.

If the cat does not approach but shows no other behaviours listed in chart 3, proceed to approach the cat and move on to step 4.

4. While talking to the cat, begin to gently stroke the cat along the head, back, and sides. The cat:

	Tick all that apply
Has large, dilated pupils	
Has its whiskers held back close to its face	
Swishes its tail quickly from one side to another	
Holds its head position low (on the plane of the body or lower)	
Crouches on all four paws, with its body near to the ground	
Retreats	
Hisses/Growls*	
Hides *	
Flattens ears *	
Strikes/attempts to strike hand – fast movement with claws outstretched *	
Bites/attempts to bite hand – fast movement, if the cat makes contact the bite is of fair force (enough to hurt or mark observer) *	

Other observations____

____

5. Move away from the cat and move a piece of string along the floor slowly to initiate play (or use other toy as needed). The cat:

	Tick all that apply
Avoids eye contact	
Watches with no approach	
Has its whiskers held back close to its face	
Retreats	
Hisses/Growls*	
Hides *	
Flattens ears *	

Other observations____

____

6. Call the cat again until it approaches or approach it slowly yourself. Begin to stroke the cat again and if the cat is calm, pick up the cat gently and cradle it against your chest for a period of 4 seconds (if the cat is not calm do not proceed to picking the cat up – if this is the case please make a note in ‘other observations’). The cat:

	Tick all that apply
Has large, dilated pupils	
Has its whiskers held back close to its face	
Swishes its tail quickly from one side to another	
Hisses/Growls*	
Flattens ears *	
Struggles to escape*	
Strikes/attempts to strike hand – fast movement with claws outstretched *	
Bites/attempts to bite hand – fast movement, if the cat makes contact the bite is of fair force (enough to hurt or mark observer) *	

Other observations____

____
